# Small Molecule Cocktail DLC79 Suppresses Gliomagenesis by Activating Ascl1 and Remodeling Transcriptome

**DOI:** 10.3390/cells15020211

**Published:** 2026-01-22

**Authors:** Chuxiao Mao, Zhancheng Deng, Zhuming Chen, Lirong Huang, Caiyun Wang, Gong Chen, Qingsong Wang

**Affiliations:** 1 Key Laboratory of CNS Regeneration (Ministry of Education), Guangdong-Hongkong-Macau Institute of CNS Regeneration, Jinan University, Guangzhou 510632, China; maocx99@163.com (C.M.); dann392@163.com (Z.D.); chenzj33@163.com (Z.C.); hlr_1209@163.com (L.H.); wangcaiyun0409@163.com (C.W.); 2 Administrative Committee of Guangzhou-Hong Kong Intelligent Manufacturing Cooperation Zone, Guangzhou Development District, Guangzhou 510730, China; 3 Orthopaedic Center, The Second Affiliated Hospital of Guangdong Medical University, Guangdong Medical University, Zhanjiang 524000, China; 4 State Key Laboratory of Bioactive Molecules and Druggability Assessment, Guangdong Basic Research Center of Excellence for Natural Bioactive Molecules and Discovery of Innovative Drugs, Jinan University, Guangzhou 510632, China; 5 Chinese Medicine Guangdong Laboratory, Hengqin 519031, China

**Keywords:** glioblastoma, small molecules, pharmacological reprogramming, Ascl1, tumorigenicity suppression

## Abstract

**Highlights:**

**What are the main findings?**

- A small molecule cocktail DLC79 (DAPT, LDN193189, CHIR99021, I-BET762, and Isx9) activates endogenous Ascl1 as a key drug target and remodels transcriptional identity, inducing the pharmacological reprogramming of glioma cells to neuron-like cells.
- DLC79 suppresses oncogenic behaviors (proliferation, migration, and invasion) and tumorigenicity in vivo.

**What are the implications of the main findings?**

- By suppressing tumorigenicity while promoting neuronal differentiation, this approach may open new avenues for glioma therapy.
- As a small-molecule-based cocktail, DLC79, exemplifies a feasible pharmacological strategy for reprogramming-based therapy, contributing to drug discovery.

**Abstract:**

Glioblastoma (GBM) remains incurable due to its invasive growth and therapeutic resistance. While the neurogenic transcription factor-mediated reprogramming of glioma cells has been reported, pharmacological reprogramming offers a promising alternative due to its potential advantages for clinical translation. Using phenotype-driven screening, we identified a multi-target small-molecule cocktail DLC79 (DAPT, LDN193189, CHIR99021, I-BET762, and Isx9) that effectively reprograms human glioma cells into neuron-like cells by activating endogenous *ASCL1* (174.4-fold) and remodeling the transcriptional landscape. This conversion led to the strong upregulation of neuronal markers (e.g., *MAP2* and *GAD67*) and suppression of glial identity. Functionally, DLC79 treatment inhibited glioma malignancy in vitro, impairing proliferation, migration, invasion, and clonogenicity. In a subcutaneous xenograft model, brief pretreatment with DLC79 significantly attenuated the tumorigenic potential of glioma cells, reducing tumor bioluminescence by 56% and tumor mass by 47%. Our study establishes pharmacological reprogramming as a promising anti-glioma strategy that leverages neuronal conversion to reduce oncogenic properties, thereby initiating a novel therapeutic paradigm.

## 1. Introduction

Glioblastoma (GBM) is the most common and malignant primary brain tumor in adults, characterized by relentless proliferation, diffuse infiltration into surrounding brain tissue, and profound resistance to conventional therapies. The current standard treatment, which includes maximal safe surgical resection followed by radiotherapy and concomitant and adjuvant temozolomide (TMZ) chemotherapy, provides only a marginal survival benefit, with a median survival of approximately 15–17 months [1,2]. The inherent cellular and molecular heterogeneity of GBM, along with the presence of a robust blood–brain barrier, contributes significantly to treatment failure and the inevitable recurrence of the tumor [3]. The conventional approach to glioma management aims to kill or remove tumor cells. This strategy often results in damage to healthy brain tissue and is associated with severe side effects [4,5].

Pharmacological reprogramming has emerged as a paradigm-shifting alternative approach. By exploiting tumor cells’ plasticity, this strategy aims to pharmacologically convert malignant cells into non-proliferative, neuron-like cells—thereby ablating oncogenicity while potentially restoring neural architecture [6,7,8]. This concept builds upon studies demonstrating that the forced expression of neural transcription factors (TFs), such as Ascl1, Neurog2, NeuroD1, and NeuroD4, can induce glioma cell differentiation and suppress proliferation [9,10,11,12,13]. These TFs remodel cell fate through epigenetic modifications and the modulation of key developmental pathways like Notch, Wnt, and BMP [14,15,16,17,18,19]. Critically, small molecules present distinct pharmacological advantages over genetic TF delivery, offering precise temporal control, tunable dosing, multi-target engagement, and reduced immunogenicity [20,21]. We previously demonstrated that multi-target pharmacological agents can effectively convert astrocytes into neurons by modulating similar signaling pathways such as Notch, Wnt, and BMP [22,23]. Here, we extend this pharmacological approach to GBM, hypothesizing that modulating gliomagenic pathways with small molecules could induce therapeutic reprogramming.

This study identifies the multi-target small-molecule cocktail DLC79 (DAPT, LDN193189, CHIR99021, I-BET762, and Isx9), which pharmacologically reprograms glioma cells into GABAergic neurons through the activation of endogenous neural transcription factors such as Ascl1. DLC79 concurrently suppresses malignant phenotypes such as proliferation, migration, invasion, and clonogenicity, establishing a dual mechanism strategy with significant implications for GBM treatment.

## 2. Materials and Methods

### 2.1. Glioma Cell Culture and Neuronal Cell Induction

Human glioblastoma cell line U-251 MG (RRID: CVCL_0021, U251) was purchased from Wuhan Procell Life Science & Technology Co., Ltd. (Wuhan, China). U-87 MG-Luc (RRID: CVCL_C8WM, U87-Luci) was purchased from the German Collection of Microorganisms and Cell Cultures GmbH (DSMZ, Braunschweig, Germany). They were cultured in DMEM medium supplemented with 10% fetal bovine serum (FBS; Gibco/Thermo Fisher Scientific, Waltham, MA, USA), passaged at 90% confluence using 0.05% trypsin (Life Technology/Thermo Fisher Scientific, Waltham, MA, USA) and reseeded at a 1:5 ratio.

For neuronal induction, cells were plated on 0.1% gelatin-coated coverslips (10,000 cells/coverslip). At 80% confluence, the medium was replaced with induction medium (DMEM/F12, 0.4% B27, 0.8% N2) with small molecules (all the screened small molecules are in Table S2) or 0.05% Dimethylsulfoxide (DMSO) vehicle control for 6 days, then switched to neuronal differentiation medium (NDM) with DMEM/F12, 0.8% N2, 0.4% B27, 0.5% FBS, 1 μM Y-27632, 5 μg/mL vitamin C, 0.2% penicillin/streptomycin, 10 ng/mL Brain-Derived Neurotrophic Factor (BDNF), Glial cell line-Derived Neurotrophic Factor (GDNF), Neurotrophin-3 (NT3), and 20 ng/mL Insulin Growth Factor 1 (IGF-1). The concentrations of individual small molecules were selected based on previously reported effective doses in neural reprogramming studies and further optimized empirically during the screening process to maximize reprogramming efficiency while maintaining cell viability.

For extended culture experiments (up to 40 days), after the initial 6-day induction with small molecules or DMSO, cells were maintained in the differentiation medium with medium changes every 3–4 days.

### 2.2. Immunofluorescence Staining and Imaging

Cells were fixed in 4% Paraformaldehyde (PFA, LEAGENE, Beijing, China) for 15 min, permeabilized with 0.2% Triton X-100, blocked 3% bovine serum albumin (BSA) in phosphate-buffered saline (PBS), and incubated with primary antibodies at 4 °C overnight. After washing three times with 0.2% PBS containing Tween 20 (PBS-T), cells were incubated with fluorescence-conjugated secondary antibodies for 1 h at room temperature in the dark. The details of all the antibodies are listed in Table S1. Finally, the samples were mounted with an anti-fade mounting solution (Sigma-Aldrich, St. Louis, MO, USA) and imaged using Zeiss (Jena, Germany) confocal fluorescence microscopy.

The reprogramming efficiency, expressed as the percentage of Doublecortin (DCX)-positive (DCX^+^) cells, was quantified by the manual counting of DCX immunopositive cells and total DAPI-labeled nuclei from at least 20 randomly selected fields per condition across *n* ≥ three independent replicates. The percentage was calculated as (number of DCX^+^ cells/total number of DAPI^+^ cells) × 100%.

### 2.3. RNA Isolation, Reverse Transcription, and RT-qPCR

The total RNA of U251 cells was isolated using the TRIzol (Thermo Fisher Scientific, Waltham, MA, USA) method following the manufacturer’s protocols at day 2, 4, and 6. The RNA of each sample was reverse transcribed by the 1st Strand cDNA Synthesis Kit (Roche, Basel, Switzerland). Then, 2× SYBR Green PCR Master Mix (QIAGEN, Hilden, Germany) was used for RT-PCR. Each sample had three replicates. The primer sequences are listed as below (Table 1).

### 2.4. Cell Proliferation Assay

Cell proliferation was measured using a Cell Counting Kit-8 (CCK-8) assay. The cells were seeded in 96-well plates (4000 cells/well), treated with DLC79 or DMSO control for the indicated times (*n* = five replicates per group), and then incubated with CCK-8 reagent (GLPBIO, Montclair, CA, USA) for 1 h at 37 °C. The absorbance at 450 nm was measured using a microplate reader (Bio Tek, Winooski, VT, USA). The proliferation curve was plotted using GraphPad Prism (version 8.2.1, GraphPad Software, San Diego, CA, USA).

### 2.5. Cell Apoptosis Analysis by Annexin V/PI Staining

Cells were seeded in six-well plates and treated with indicated compounds. After 48 h, the cells were harvested with trypsin, washed twice with cold PBS, and resuspended in 100 μL of Annexin V binding buffer. The cell suspensions were incubated with 5 μL of Annexin V-FITC and 5 μL of propidium iodide (PI, 50 μg/mL) for 15 min at room temperature in the dark. Following incubation, 400 μL of binding buffer was added, and the samples were immediately analyzed by flow cytometry (Accuri C6, BD Biosciences, San Jose, CA, USA). The percentages of early apoptotic (Annexin V^+^/PI^−^) and late apoptotic/necrotic (Annexin V^+^/PI^+^) cells were quantified using FlowJo software (version 10.8.1).

### 2.6. Cell Cycle Analysis by PI Staining

Cells were plated and treated as described above. After harvesting, the cells were washed with PBS and fixed in 70% ice-cold ethanol dropwise while vortexing gently. The fixed cells were stored at −20 °C overnight, then washed and resuspended in PBS containing 50 μg/mL PI and 100 μg/mL RNase A. The samples were incubated for 30 min at 37 °C in the dark and analyzed by flow cytometry. Cell cycle distribution (G_0_/G_1_, S, and G_2_/M phases) and the sub-G_1_ apoptotic fraction were determined using FlowJo software (version 10.8.1).

### 2.7. Trans-Well Assay

Cells pretreated for 4 days were seeded in Matrigel-coated (invasion) or uncoated (migration) trans-well chambers (Corning Costar, Kennebunk, ME, USA; 8.0 μm pore size). After 24 h, the cells were fixed, stained with crystal violet, and counted (ImageJ v1.54). Triplicate experiments were performed.

### 2.8. Clonogenic Assay

The cells (300/well) were treated with half-concentration DLC79 or DMSO for 6 days, then cultured in DMSO for 4 days, and each group had three replicates. When visible colonies were formed, the cells were fixed with 4% PFA and stained with crystal violet solution. The numbers of colonies were counted using ImageJ version 1.54.

### 2.9. Single-Cell Sequencing

U251 cells treated with DLC79 or DMSO for 4 days were processed by SeekGene BioSciences (Beijing, China). Single-cell RNA-Seq libraries were prepared using SeekOne^®^ Digital Droplet Single Cell 3′ library preparation Kit (SeekGene, Beijing, China). Data were aligned to human GRCh38, analyzed with Seurat (version 4.1.1, clustering resolution: 0.3), and visualized via Uniform Manifold Approximation and Projection (UMAP). The lineage differentiation was assessed with Monocle 2 (version 2.26.0) for pseudotime trajectory analysis, employing default settings. The singleR package (version 1.8.1), with CellDex’s Human Primary Cell Atlas Data as the reference dataset. Identified differentially expressed genes (DEGs) were then subjected to Gene Ontology (GO) enrichment analysis for biological processes via Over-Representation Analysis (ORA).

### 2.10. Animals

BALB/c nude mice (aged 5–7 weeks) were procured from Guangdong Jicui Yaokang Biotechnology Co., Ltd. (Guangzhou, China). The mice were housed under specific pathogen-free (SPF) conditions (25 °C, 40–60% humidity, and 12 h light/dark cycle).

### 2.11. Subcutaneous Tumor Model Establishment

The U-87 MG-Luc cells (1.2 × 10^7^ cells/mL) were pretreated with DLC79 or 0.5% DMSO for 6 days. The mice were anesthetized with 2% isoflurane and 100 μL cell suspension was injected subcutaneously. The mice were monitored daily and euthanized upon reaching ethical limits (tumor volume ≤ 2000 mm^3^).

### 2.12. In Vivo Bioluminescence Imaging (BLI)

The mice received intraperitoneal D-luciferin potassium salt (150 mg/kg; GoldBio, St. Louis, MO, USA) and were imaged every 3–4 days using IVIS Spectrum (PerkinElmer, Waltham, MA, USA). The bioluminescent intensity (photons/second) within the tumor regions of interest (ROIs) was quantified using Living Image software (version 4.8.1).

### 2.13. Data and Statistics Analysis

Data analysis was performed using GraphPad Prism (version 8.2.1, GraphPad Software, San Diego, CA, USA). Data are represented as mean ± SEM (standard error of the mean). Student’s *t*-test and a one-way ANOVA were employed to assess the significance of differences between groups. A *p*-value of less than 0.05 was considered to indicate a statistically significant difference.

## 3. Results

### 3.1. Phenotype-Driven Discovery of DLC79: A Multi-Target Reprogramming Cocktail

**Figure 1:**
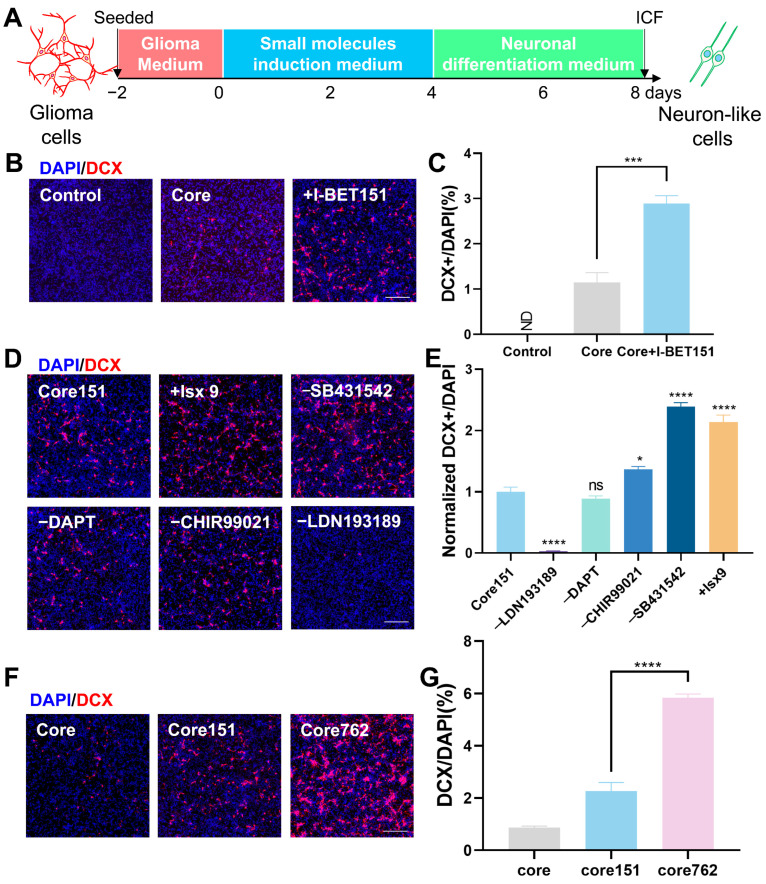
Identification of a small-molecule cocktail for reprogramming human glioma cells into neuron-like cells. (**A**) Schematic diagram illustrating the experimental process for the pharmacological reprogramming of glioma cells using the small-molecule cocktail. The arrows represent seeded cells and cells subjected to immunofluorescence staining. (**B**) Representative immunofluorescence images showing that the addition of I-BET151 enhances the generation of neuron-like cells, which express the neuronal marker DCX (red). Nuclei are counterstained with DAPI (blue). (**C**) Quantification of the reprogramming efficiency, presented as the percentage of DCX-positive cells among total DAPI-labeled cells. The ND means not detected (*n* ≥ 3). (**D**,**E**) The optimization based on the cocktail Core + I-BET151 (Core151). The addition of 10 μM Isx-9 further improved the conversion efficiency. Systematic omission of individual components (SB431542, DAPT, CHIR99021, or LDN193189) from the Core cocktail revealed that SB431542 is dispensable, whereas LDN193189 is essential for the induction process. The ns in figure (**E**) indicates no significant difference (*n* = 3). (**F**,**G**) Substitution of I-BET151 with its functional analog, I-BET762, increased the efficiency of neuronal conversion. (*n* ≥ 3). All scale bars represent 300 μm. Statistical significance was determined by one-way ANOVA with Tukey’s multiple comparisons test; * *p* < 0.05, *** *p* < 0.001, and **** *p* < 0.0001.

Building on our prior work converting astrocytes to neurons [24], we hypothesized that glioma cells, sharing glial lineage plasticity, might be pharmacologically reprogrammed into neuron-like cells by using the small-molecule approach. Based on the core rationale of simultaneously modulating key signaling pathways, including transforming growth factor beta (TGF-β), bone morphogenetic protein (BMP), Notch, Wnt, and Hedgehog, involved in neural development and cell fate determination, we constructed a small-molecule cocktail using the Core drug consisting of SB431542, DAPT, CHIR99021, and LDN193189 [24]. We subsequently screened 14 neurogenic or anti-tumor compounds (Table S2) using the process illustrated in Figure 1A. The selection of candidates for the initial screening was based on their ability to target pathways complementary to the Core. The results show that I-BET151 [25,26,27] (BET inhibitor) significantly enhanced DCX (a marker for early neuronal development)-positive cells with higher conversion efficiency compared to the Core drug (2.58% vs. 1.15%; Figure 1B,C).

In order to further improve the conversion efficiency, subsequent optimization based on cocktail Core + I-BET151 (Core151) revealed that the addition of Isx9 [28] nearly doubled the induction efficiency of DCX-positive cells (as shown in Figure 1D–F). Interestingly, the exclusion of SB431542 increased the efficiency by 2.4-fold (Figure 1D,E). Few DCX signals could be detected after the removal of LDN193189 (Figure 1D), suggesting that LDN193189 may be a critical component in the glioma cell to neuron reprogramming. Compared to I-BET151, I-BET762 [29] (advanced BET inhibitor) further elevated the conversion efficiency to 5.84% with 4 days of treatment (Figure 1G). Together, the optimized cocktail comprises the following: DAPT (5 μM), LDN193189 (1 μM), CHIR99021 (1.5 μM), I-BET762 (1 μM), and Isx9 (5 μM), briefly called DLC79 hereafter (detailed in Table 2). This represents a rationally designed, multi-target pharmacological strategy to reprogram glioma cells into neurons.

### 3.2. Essentiality of DLC79 Components for Reprogramming Efficacy

**Figure 2:**
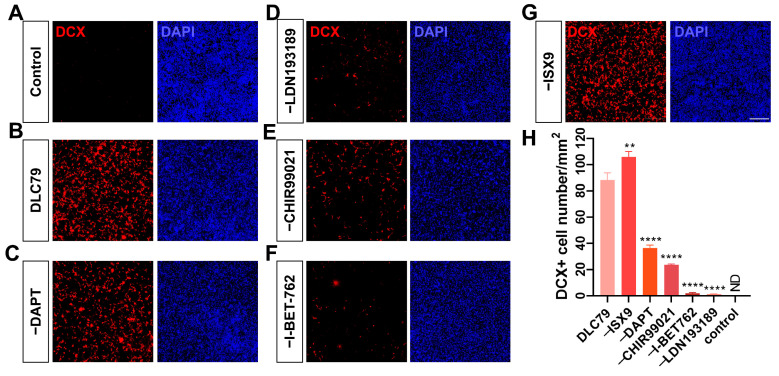
Functional screening identifies essential small molecules for reprogramming. (**A**) U251 was treated with 0.05% DMSO as the control group. DCX, red; DAPI, blue. (**B**) DLC79-induced pharmacological reprogramming as a positive control at day 10. (**C**–**G**) Removal of individual components—DAPT (**C**), LDN193189 (**D**), CHIR99021 (**E**), I-BET762 (**F**), or Isx-9 (**G**)—from DLC79 reduced the ratio of DCX-positive cell generation. (**H**) Quantification of DCX-positive cells confirms LDN193189 as essential, with I-BET762, CHIR99021, and DAPT also contributing significantly to reprogramming efficiency. The ND means not detected (*n* = 3). Scale bars: 300 μm. ** *p* < 0.01 and **** *p* < 0.0001; one-way ANOVA with Tukey’s multiple comparison test.

Component exclusion assays demonstrated non-redundant roles in DLC79. The omission of the BMP pathway inhibitor LDN193189 or the BET protein inhibitor I-BET762 abolished reprogramming (Figure 2D,F). The removal of DAPT or CHIR99021 reduced DCX^+^ cells by 64% and 77%, respectively (Figure 2C,E,H). The omission of Isx9 increased the DCX^+^ cells, while the total glioma cell number also increased compared to the control group (Figure 2G,H). Ultimately, we concluded that the optimal combination remains as DLC79, which achieves 88 DCX-positive cells per mm^2^ (Figure 2H). Although the conversion number appeared to be low, the other non-converting glioma cells were affected by the small molecules as well; see the further investigation below.

### 3.3. DLC79 Drives Pharmacological Reprogramming of Neuronal Identity

**Figure 3:**
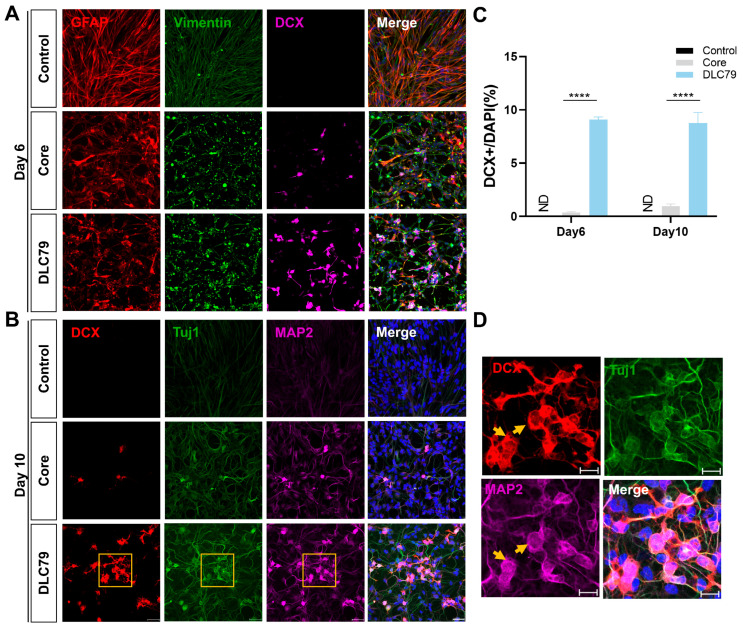
DLC79 induces pharmacological reprogramming of neuronal identity. (**A**) Immunofluorescence analysis at day 6 shows that DLC79 generates more DCX^+^ (violet) neuron-like cells than the Core cocktail or DMSO control. Reprogrammed cells lose expression of the astrocyte marker GFAP (red) and mesenchymal marker vimentin (green). (**B**,**D**) By day 10, induced cells exhibit stronger neuronal morphology and co-express immature markers DCX (red) and TUJ1 (green), alongside the mature marker MAP2 (violet). Panel (**D**) shows a magnified view of the boxed region in (**B**), with arrows indicating triple-positive cells. (**C**) Quantification confirms that DLC79 significantly enhances DCX^+^ cell conversion compared to the Core cocktail. The ND means not detected. Scale bars: 300 μm (**A**,**B**); 50 μm (**D**). **** *p* < 0.0001 by one-way ANOVA with Tukey’s multiple comparisons test.

Next, we further examined the cell identity after DLC79 treatment using a series of neuronal markers. After six days of DLC79 treatment, the induced DCX-positive cells exhibited a bipolar neuron-like morphology and lacked co-labeling with the glial cell marker GFAP and the epithelial–mesenchymal transition (EMT) marker Vimentin (Figure 3A). Subsequently, upon switching to a neural differentiation medium, an increased number of DCX-positive neuron-like cells were observed by Day 10, which co-expressed with the neuronal markers Tuj1 and MAP2 (Figure 3B,D).

In comparison to the initial Core drug cocktail, our refined multi-target cocktail, DLC79, markedly enhanced the number and induction efficiency of DCX-positive neuron-like cells. DLC79 treatment for 6 days achieved a peak conversion efficiency of approximately 9.09% for DCX^+^ neuron-like cells. Even after switching to the factor-enriched neuronal differentiation medium (NDM) from day 6, the conversion efficiency driven by DLC79 was not significantly enhanced (Figure 3C). This result demonstrates that, unlike the Core cocktail, DLC79 exhibits a lower dependency on exogenous differentiation cues for inducing neuronal conversion.

### 3.4. Single-Cell Transcriptomics Reveals Reprogramming Trajectories

**Figure 4:**
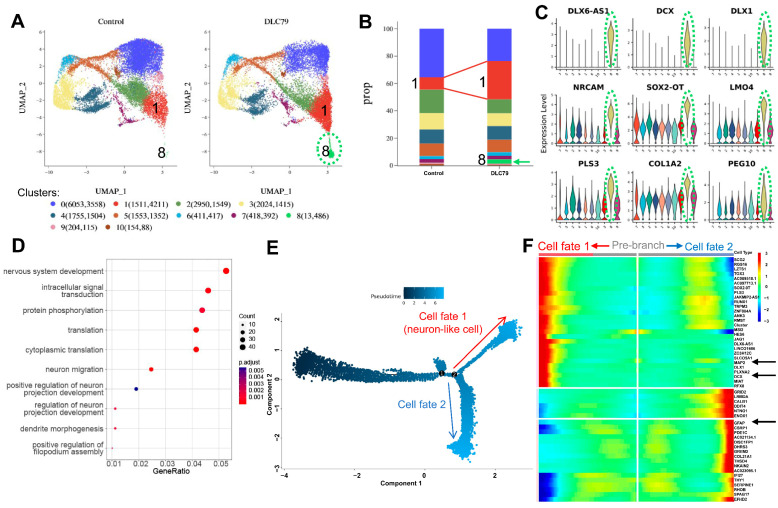
Single-cell transcriptomics reveal DLC79-driven neuronal reprogramming trajectory. (**A**) UMAP visualization of single-cell transcriptomes from the control and DLC79-treated U251 cells. A new neuronal cluster (cluster 8; green ellipse) emerges following DLC79 treatment. (**B**) Cellular composition analysis shows a marked expansion of cluster 1 (neural development related) and the emergence of cluster 8 (indicated by the green arrow). (**C**) Violin plots display expression distribution of top neuronal genes (e.g., *DCX* and *DLX1*) in cluster 8 (green dash ellipse). (**D**) GO enrichment analysis of differentially expressed genes. Bubble color indicates statistical significance (−log_10_(adjusted *p*-value)); size reflects number of genes per term. (**E**) Pseudotime trajectory analysis reveals two distinct cell fates after DLC79 treatment, with one branch progressing toward neuronal identity. Numbers 1 and 2 indicate the two divergent cell roots. (**F**) Heatmap shows DEG expression dynamics at branching point 2 in (**E**) on the pseudotime trajectory. The arrows highlight key neurodevelopmental (e.g., *DCX* and *MAP2*) are upregulated in fate 1 (red arrow), while glial (e.g., *GFAP*) identity is retained in fate 2.

To elucidate the mechanism underlying DLC79-induced neuronal reprogramming, we performed single-cell RNA sequencing on U251 cells after 4 days of treatment. UMAP analysis partitioned 32,133 cells into 11 distinct clusters (Figure 4A). DLC79 treatment markedly reduced tumor-related clusters (cluster 0, 2, and 3), which expressed genes linked to the tumor immune microenvironment (e.g., *NPY1R* and *MEST*), extracellular matrix (*CCN1* and *RUNX2*), and proliferation-associated histones (*HIST1H4C*). Conversely, clusters 1 and 8 were substantially expanded. Cluster 1, from 1511 to 4211, showed enrichment for neurodevelopmental genes (*RGS16* and *SCG2*), while cluster 8—increasing 37-fold after treatment—displayed strong induction of neuronal markers *DCX*, *DLX1*, and *NRCAM* (Figure 4B,C and Table S3). Additionally, high expression levels of *SOX2-OT* (SOX2 overlapping transcript, lncRNA) and *LMO4* (LIM domain-only 4) related to neurogenesis and neuron development were observed in these cells (Figure 4C).

Gene Ontology (GO) analysis within cluster 1 confirmed enrichment in nervous system development, neuron migration, and axon projection (Figure 4D). This analysis revealed that the DEGs are predominantly associated with key biological processes such as nervous system development, intracellular signal transduction, protein phosphorylation, translation, cytoplasmic translation, neuron migration, and the positive regulation of neuron projection development (Figure 4D).

Pseudotime trajectory analysis further revealed two divergent cell fates: one progressing toward a neuron-like state (cell fate 1), and the other retaining glial identity (cell fate 2, Figure 4E). The heatmap of DEGs at branch point 2 highlighted the gene expression patterns distinguishing these two fates, with neuro-related genes such as *DCX*, *MAP2*, and *DLX1* being highly expressed in the neuron-like cells, and GFAP expression being low (Figure 4F). These results demonstrate that DLC79 drives transcriptional remodeling toward a neuronal gene program, effectively suppressing glioma cell identity.

### 3.5. Pharmacodynamic Profile of DLC79-Induced Reprogramming

**Figure 5:**
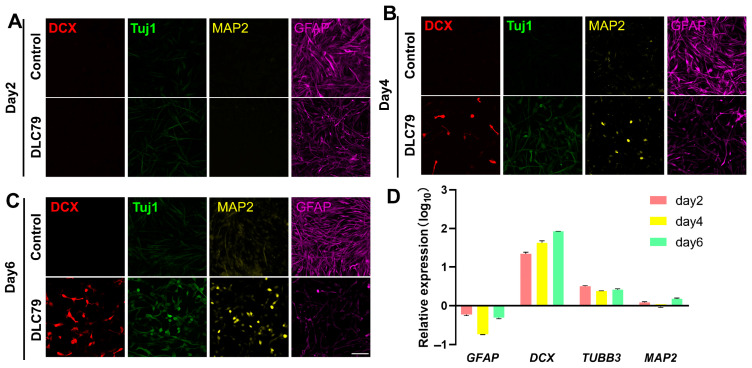
DLC79 induces progressive neuronal marker expression and suppresses astrocytic identity. (**A**–**C**) Immunofluorescence images of U251 cells treated with DLC79 on day 2, day 4, and day 6, demonstrating the time-dependent upregulation of neuronal markers (DCX, TUJ1, and MAP2) and the downregulation of the astrocytic marker GFAP. Scale bar: 100 μm. (**D**) RT-qPCR analysis confirms the transcriptional activation of neuronal genes (*DCX*, *TUBB3*, and *MAP2*) and repression of *GFAP* during treatment. Data are normalized to the DMSO control and presented as mean ± SEM; *n* = 3.

We assessed neuronal reprogramming over the short-term (days 2, 4, and 6) and extended (up to day 40) the culture periods by immunofluorescence for Tuj1, MAP2, DCX, and GFAP. DCX^+^ cells emerged at day 4 and increased markedly by day 6 (Figure 5A–C). Tuj1 and MAP2 expression also intensified over time, while GFAP declined. At day 24, a subset of MAP2^+^ cells co-expressed GABA, suggesting an inhibitory neuronal phenotype; other subtype markers were undetected (Figure S1A).

The qPCR analysis revealed rapid upregulation of neuronal genes: *DCX* increased nearly 20-fold by day 2 and over 80-fold by day 6; *TUBB3* peaked at 3-fold on day 2 and remained elevated. *MAP2* showed modest increase (1.5-fold) only on day 6. In contrast, *GFAP* expression dropped sharply to less than 0.2 times of the control by day 4 and partially recovered by day 6 (Figure 5D). These results, consistent with the protein expression patterns, underscore the upregulation of neural-related genes and the downregulation of glia-related genes during DLC79 treatment.

After 24 days, many cells retained DCX expression with elongated neurites and enhanced MAP2/Tuj1 co-localization, but NeuN remained undetected (Figure S1B). By day 40, RT–qPCR showed that *NeuroD1* and *Neurog2* had been downregulated to levels comparable to the control, while *ASCL1* remained elevated (nearly 10-fold). The expression of *MAP2*, which had shown only a modest (1.5-fold) increase at day 6 of induction, gradually amplified to approximately 3-fold relative to the control. *NEUN* was undetectable in both groups, indicating incomplete neuronal maturation under these culture conditions (Figure S1C).

### 3.6. DLC79 Triggered the Activation of Neuronal Transcription Factors

To investigate the transcriptional mechanisms underlying DLC79-induced neuronal reprogramming in glioma cells, we performed single-cell regulatory network inference and clustering (SCENIC) analysis [30]. This revealed altered activity of key transcription factors (TFs): *POU3F2*, *SOX9*, and *STAT2* were downregulated, while *RUNX1*, *SOX4*, and *KLF12* were upregulated in DLC79-treated cells (Figure S2A), which was consistent with the RT–qPCR results (Figure S2B). Notably, DLC79 activated the DLX family (*DLX1* and *DLX6*) and bHLH family (*Ascl1*) TFs.

**Figure 6:**
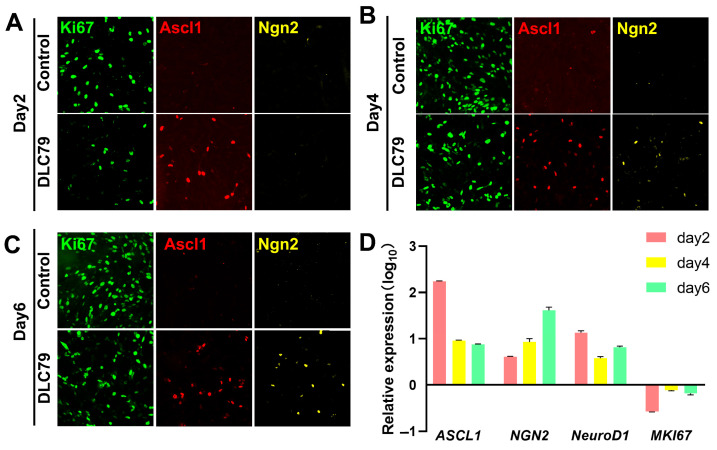
DLC79 activated neural transcription factors and suppresses proliferation. (**A**–**C**) Immunofluorescence images of U251 cells treated with DLC79 for 2–6 days, showing decreased Ki67 and induced expression of neural transcription factors ASCL1 and Neurogenin 2 (Ngn2). Scale bar: 100 μm. (**D**) RT-qPCR analysis confirms the downregulation of *MKI67* and upregulation of *ASCL1*, *NEUROG2*, and *NEUROD1* during reprogramming. Data are normalized to the DMSO control and shown as mean ± SEM (*n* = 3).

KI67 protein levels were downregulated during treatment with DLC79, with the most pronounced suppression observed on day 2, after which KI67-positive cell counts gradually returned to levels comparable to the control group (Figure 6A–C). This could be attributed to contact inhibition. Notably, the neural transcription factor Ascl1 was activated on day 2 (Figure 6A). On day 4, a subset of cells expressed the neural transcription factor Neurog2 (Figure 6B), with a higher expression on day 6 (Figure 6C).

The gene expression pattern of *MKI67* in the DLC79-treated group mirrored the staining results, with expression being suppressed relative to the control group (Figure 6D). Notably, *MKI67* expression showed a transient suppression at day 2 followed by a recovery towards baseline by day 6, suggesting a dynamic or heterogeneous response to the reprogramming cue. *Ascl1* exhibited the most profound upregulation, peaking at over 170-fold on day 2 before declining to 8-fold by day 6. *Neurog2* expression rose steadily, reaching 42-fold by day 6. This rapid, potent induction of Ascl1 aligns with its established role as a master regulator capable of driving neuronal conversion, suggesting it is a key pharmacological mediator of DLC79′s effects (Figure 6D).

### 3.7. DLC79 Reduces Malignant Phenotypes of Glioma Cells In Vitro

**Figure 7:**
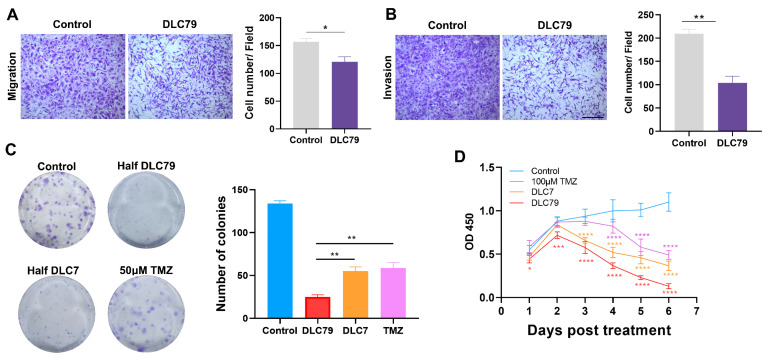
DLC79 reduces malignant phenotypes of glioma cells in vitro. (**A**,**B**) Trans-well assays show that DLC79 significantly impairs the migration (**A**) and invasion (**B**) of U251 cells. (**C**) Colony formation is strongly inhibited by DLC79, with greater efficacy than DLC7 or 100 μM temozolomide (TMZ). (**D**) CCK-8 assay confirms that DLC79 potently suppresses cell proliferation, outperforming both DLC7 and TMZ. Scale bars are shown in panels; *n* = 3. * *p* < 0.05, ** *p* < 0.01, *** *p* < 0.001, and **** *p* < 0.0001; Student’s *t*-test (**A**,**B**), one-way ANOVA (**C**), or two-way ANOVA (**D**) with appropriate post hoc tests.

We evaluated the anti-tumor effects of DLC79 using functional assays. Trans-well experiments showed that DLC79 significantly impeded the migration and invasion of U251 and U87-Luci cells (Figure 7A,B and Figure S3A,B). DLC79 decreased migrating, from 156.6 ± 8. 5 in the control group to 120.5 ± 13.4 in the group. Similarly, the total number of invading cells was reduced from 209.5 ± 13.2 to 103.8 ± 20.4 cells in the DLC79 group. Colony formation was also strongly inhibited: DLC79 reduced the number of U251 colonies by over 75% compared to the controls (25.0 ± 3.6 vs. 134.0 ± 4.3 in control), an effect dependent on the full combination, outperforming both the incomplete cocktail (DLC7) and temozolomide (Figure 7C). A similar suppressive effect was observed in U87-Luci cells (Figure S3C).

CCK-8 assays revealed that DLC79 strongly inhibited proliferation, with a 38.4% reduction by 48 h and 88.2% suppression on day 6, outperforming 100 μM TMZ (55.5% inhibition) (Figure 7D). Proliferation arrest was associated with the downregulation of MKI67 (Figure 6D). U87-Luci cells also exhibited sustained proliferation arrest under DLC79 treatment (Figure S3D).

Together, these data demonstrate that DLC79 effectively curbs glioma aggressiveness by inhibiting migration, invasion, clonogenicity, and proliferation, with converted cells exhibiting immature neuronal features.

### 3.8. DLC79 Treatment Attenuates Tumor Growth In Vivo

**Figure 8:**
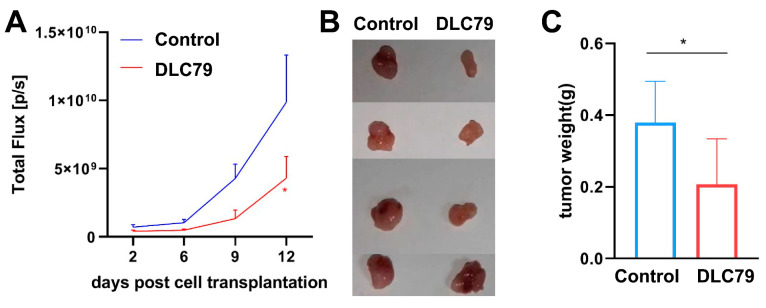
DLC79 pretreatment decreased tumorigenicity of glioma cells in vivo. (**A**) Quantification of bioluminescent radiance from day 2 to day 12 shows significantly reduced tumor viability in the DLC79 group (two-way ANOVA with Sidak’s multiple comparisons; * *p* < 0.05; and *n* = 4). (**B**) Representative images of dissected subcutaneous tumors from each group at endpoint (day 12). (**C**) Tumor weights confirm that DLC79-pretreated cells formed significantly smaller masses than controls (Student’s *t*-test; * *p* < 0.05). All data represent mean ± SEM; *n* = 4.

To evaluate the persistent, cell-intrinsic effect of DLC79 on the tumor-initiating capacity, we subcutaneously implanted U87-Luci cells that had been pretreated in vitro with DLC79 or DMSO for 6 days into nude mice. Quantitative analysis showed a 56% reduction in BLI signal intensity in the DLC79 group (4.3 × 10^9^ p/s vs. 9.9 × 10^9^ p/s in controls) by day 12 (Figure 8A). The excised tumors from the DLC79 group were visibly smaller and demonstrated a 47.4% reduction in average weight (0.20 g vs. 0.38 g in controls) (Figure 8B,C). No significant body weight loss or systemic toxicity was observed. These results indicate that brief preconditioning with DLC79 effectively attenuates the tumorigenic potential of glioblastoma cells in vivo. Furthermore, the pretreated cells were not overtly toxic, as evidenced by the absence of significant body weight loss or systemic illness in mice compared to the controls.

## 4. Discussion

Building upon our demonstration of pharmacological reprogramming in astrocytes, this study establishes that glioma cells are similarly amenable to small molecule-induced neuronal conversion. We identified the multi-target cocktail DLC79—comprising DAPT, LDN193189, CHIR99021, I-BET762, and Isx9—as a potent multi-target inducer of neuronal reprogramming in human glioma cells. LDN193189 proved essential for reprogramming efficacy, while SB431542 reduced efficiency. SB432542, a TGF-β pathway inhibitor targeting the Alk 4, Alk 5, and Alk 7 receptors, is often paired with LDN193189, which targets the Alk 2 and Alk 3 receptors, to serve as dual SMAD inhibitors [18,31,32,33]. It has been shown that these two molecules may elicit opposing serotonergic differentiation effects during the in vitro induction of mouse embryonic stem cells [34]. This divergence may result from their complex interactions with additional pathways or kinases [35], reflecting complex pathway interactions during glial-to-neuronal transition. Isx9 was retained despite decreasing the DCX^+^ cell number due to its critical neuro-inductive properties [36], exemplifying rational polypharmacology optimization.

### 4.1. Strengths

DLC79 triggered Ascl1-dependent transcriptional remodeling, upregulating neuronal markers (*DCX* increased 80-fold) while suppressing glial identity (*GFAP* decreased 80%). Single-cell RNA sequencing confirmed the induction of a GABAergic neuronal trajectory, consistent with ASCL1′s role in inhibitory neuron development. With extended culture, the induced neuron-like cells exhibited continuous neurite elongation and expressed the inhibitory neuronal marker GABA, aligning with the finding that Ascl1 predominantly promotes the generation of GABAergic neurons in the reprogramming of human glioma cells [37]. The resulting neuron-like cells exhibited extended neurites and expressed GABA, supporting a functional shift toward a neuronal phenotype.

Functionally, DLC79 suppressed malignant behaviors—proliferation, migration, invasion, and clonogenicity—in glioma models. Notably, DLC79 treatment reduced in vivo tumor growth by 56%, indicating a reduction in tumorigenicity through pharmacological reprogramming.

### 4.2. Limitations and Future Directions

This study has several limitations that should be considered. First, while systemic administration after tumor transplantation represents the gold standard for evaluating therapeutic efficacy, our subcutaneous model involving the implantation of pretreated cells was primarily designed to assess the intrinsic and persistent changes induced by DLC79, rather than to directly simulate a clinical treatment regimen. Second, this work serves as a proof-of-concept study to establish the initial efficacy and potential mechanism of DLC79. The use of established cell lines, rather than patient-derived cells or orthotopic models, limits the generalizability of our findings, and validation in more representative and clinically relevant models is warranted. Third, the observation of incomplete tumor ablation suggests that a subpopulation of cells may exhibit intrinsic resistance, or that the drug-induced suppression of the oncogenic phenotype is not fully sustained in vivo. Fourth, while our single-cell RNA sequencing data revealed a transcriptional shift towards a neuronal program, the absence of complementary epigenomic data (e.g., ATAC-seq) limits our ability to fully characterize the chromatin remodeling dynamics underlying this reprogramming. Future work integrating multi-omics approaches will provide a more comprehensive understanding of the epigenetic remodeling driven by DLC79. The initial interpretation of DLC79′s mechanism was limited by the lack of direct analysis of the cell cycle and apoptosis. To address this, we performed the recommended flow cytometry analyses. The results confirmed that DLC79 treatment induced significant apoptosis (increased Sub-G1 and Annexin V+ populations) in the treated cells (Figure S4). This demonstrates that the suppression of malignancy involves not only phenotypic modulation but also the direct induction of cell death in a subset of the population. Future studies should investigate the molecular pathways linking the activated neurogenic program (e.g., ASCL1 induction) to the cell fate decision across glioma models with different genetic backgrounds.

Furthermore, the observed transient suppression of the proliferation marker Ki-67 (*MKI67*) in vitro, which recovered partially by day 6 (Figure 6D), may appear contradictory to the sustained 56% reduction in tumor growth in vivo. This apparent discrepancy can be reconciled by considering several non-exclusive mechanisms. First, the initial potent arrest likely reflects the direct anti-proliferative effect of the cocktail, captured by the CCK-8 assay (Figure 7D). The subsequent recovery may stem from cellular heterogeneity, where a subpopulation adapts or resists full cell cycle exit, and from contact inhibition in confluent cultures. Crucially, the in vivo tumorigenicity assay measured the functional outcome of a 6-day pretreatment. This pretreatment establishes a persistent reprogrammed transcriptional state (Figure 4) that compromises the long-term tumor-initiating capacity, independent of the instantaneous Ki-67 level at the implantation timepoint. Thus, the in vivo efficacy reflects a stable impairment of oncogenic potential induced by the reprogramming event, rather than a mere continuation of the acute cytostatic state observed in vitro. This also explains that while the in vitro neuronal conversion efficiency of DLC79 is ~9%, the observed 56% reduction in in vivo tumor growth is likely a composite effect. This includes the direct loss of proliferative capacity in the converted neuron-like subset, coupled with a broader DLC79-induced suppression of oncogenic behaviors (proliferation, migration, etc.) across the treated cell population, as demonstrated by our functional assays. This suggests that DLC79 may induce a stable, less aggressive cellular state, which in itself constitutes a valuable therapeutic strategy.

Future studies aimed at in vivo therapeutic application will need to address BBB (blood–brain barrier) penetration [38] and potential systemic toxicity. Reformulation (e.g., using nanocarriers) and the identification of the minimal effective component combination [38,39,40] will be critical next steps. To enhance efficacy, future studies could explore combining DLC79 with established clinical modalities. Finally, despite the translational challenges related to blood–brain barrier penetration and sustained drug delivery, these may be addressed by integrating DLC79 with advanced carriers (e.g., liposomes, exosomes, or novel nanocarriers) or through specific chemical modifications to improve its pharmacokinetic profile. These approaches represent the key foci for future optimization.

## 5. Conclusions

In conclusion, our research demonstrates that the DLC79 multi-target pharmacological agent effectively induces glioma cells to adopt neuronal characteristics and reduces their oncogenic behavior, primarily through the chemical activation of the key neural transcription factor Ascl1. The DLC79 effectively reduces the tumorigenicity of U87-Luci glioblastoma cells in vivo, highlighting the potential of pharmacological reprogramming as a paradigm-shifting anti-glioblastoma therapy. This work opens avenues for targeting cellular plasticity in glioma through small molecule-driven transcriptional remodeling.

## 6. Patents

A patent application related to the cocktail of small molecule compounds and their use in glioma treatment in this manuscript has been filed in China by Gong Chen, Chuxiao Mao, Qingsong Wang, and Zhuming Chen, Jinan University. The allocation number is 202410742908.3.

## Figures and Tables

**Table 1:** Primer sequences for real-time PCR.

Primers	FP 5′ → 3′	RP 5′ → 3′
*ASCL1*	CGCGGCCAACAAGAAGATG	CGACGAGTAGGATGAGACCG
*DCX*	TTCAAGGGGATTGTGTACGCT	GTCAGACAGAGATCGCGTCAG
*GFAP*	CTGCGGCTCGATCAACTCA	TCCAGCGACTCAATCTTCCTC
*MAP2*	TGGTGCCGAGTGAGAAGAAG	AGTGGTTGGTTAATAAGCCGAAG
*TUBB3*	GGCCAAGGGTCACTACACG	GCAGTCGCAGTTTTCACACTC
*NeuN*	GCCTCGCCTTTGCCGAT	AGGTAGTCAGTCAGGTCCCG
*Neurog2*	AGGAAGAGGACGTGTTAGTGC	GCAATCGTGTACCAGACCCAG
*NeuroD1*	CCTGCAACTCAATCCTCGGA	GGCATGTCCTGGTTCTGCTC
*ACTB*	CATGTACGTTGCTATCCAGGC	CTCCTTAATGTCACGCACGAT

**Table 2:** Targets, chemical structure, functions, and final concentrations of the DLC79 small-molecule cocktail.

Component	Chemical Structure	Target/Pathway	Final Concentration
DAPT	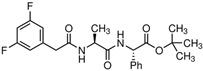	γ-secretase/Notch inhibitor	5 μM
LDN193189	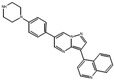	BMP receptor inhibitor	1 μM
CHIR99021	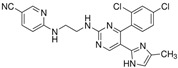	GSK-3β inhibitor/Wnt activator	1.5 μM
I-BET762	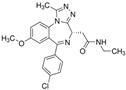	BET bromodomain inhibitor	1 μM
Isx9	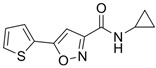	Neurogenic small molecule	5 μM

## Data Availability

All data and the original code generated for analysis will be made available upon reasonable request.

## Supplementary Materials

The following supporting information can be downloaded at: https://www.mdpi.com/article/10.3390/cells15020211/s1, Figure S1: DLC79 converted the glioma cells into inhibitory neuron cells. (A) Immunofluorescence staining images of the neuronal subtype markers GABA and MAP2 in U251 cells induced by DLC79 for 24 days. (B) Immunofluorescence staining images of the neuronal markers Tuj1, MAP2, and DCX in U251 cells induced by DLC79 for 24 days. Scale bar 100 μm. (C) The gene expression in U251 cells induced by DLC79 for 40 days compared to the control group. The ns indicates no significant difference. Data are represented as mean ± SEM. **** *p* < 0.0001, Student’s *t*-test, *n* = 3; Figure S2: DLC79 triggered the activation of neuronal transcription factors. (A) Single-cell regulatory network inference and clustering (SCENIC, version 1.1.2.1) results of the DLC79-treated group and the control group. Transcription factors highly expressed in the control group are indicated within the red boxes, while those upregulated after DLC79 treatment are marked with green boxes. Black arrows highlight key neuronal transcription factors associated with GABAergic neuron differentiation. The arrows on the left indicate highly expressed transcription factors in cluster 1 and cluster 8, respectively. (B) Quantitative PCR results of the relevant transcription factors shown in (A), presented as fold changes in the DLC79-treated group relative to the control group after 4 days of treatment. * *p* < 0.05, Student’s *t*-test, *n* = 3; Figure S3: DLC79 abrogates malignant features of U87-Luci cells. Representative pictures and quantification data showing U87-Luci migration reduced after DLC79 treatment. Scale bar. ** *p* < 0.01. (B) Representative pictures and quantification data showing U87-Luci invasion reduced after DLC79 treatment. Scale bar. *** *p* < 0.001. (C) Colony formation assay and quantification data showing DLC79 treatment inhibits colony formation ability of U87-Luci followed by DLC7 and 100 μM TMZ as a control. One-way ANOVA with Tukey’s multiple comparisons. Data are represented as mean ± SEM, * *p* < 0.05. (D) Quantitative data of CCK-8 assay showing DLC79 treatment inhibits glioma cell proliferation with a lowest OD450 value followed by DLC7 and 100 μM TMZ as a control. Two-way ANOVA with Tukey’s multiple comparisons. Data are represented as mean ± SEM, * *p* < 0.01, *** *p* < 0.001, **** *p* < 0.0001; Figure S4: Changes in apoptosis and cell cycle on Day 2 of DLC79 treatment in U251 cells. (A) The Annexin V/PI staining to quantify apoptosis, results showing that DLC79 treatment significantly increased the early apoptosis rate to 31.2%, compared to 12.8% in the DMSO control group. (B) DLC79 treatment induced a marked increase in the Sub-G1 population in DLC79 17.5%, while 7.42% in control; Table S1: The targets and functions of the screened small molecules; Table S2: The targets and functions of the screened small molecules; Table S3. Top 3 highly expression gene along significantly changed clusters in single cell sequence. References [41,42,43,44,45,46,47,48,49] are cited in Supplementary Materials.

## Abbreviations

The following abbreviations are used in this manuscript:

BDNF	Brain-Derived Neurotrophic Factor
BLI	Bioluminescence Imaging
BMP	Bone Morphogenetic Protein
CCK-8	Cell Counting Kit-8
DCX	Doublecortin
DEGs	Differentially Expressed Genes
DLC79	DAPT (5 μM), LDN193189 (1 μM), CHIR99021 (1.5 μM), I-BET762 (1 μM), and Isx9 (5 μM)
DMSO	Dimethylsulfoxide
EMT	Epithelial–Mesenchymal Transition
GBM	Glioblastoma
GDNF	Glial cell line-Derived Neurotrophic Factor
GFAP	Glial Fibrillary Acidic Protein
GO	Gene Ontology
IGF-1	Insulin Growth Factor 1
MAP2	Microtubule-Associated Protein 2
NDM	Neuronal Differentiation Medium
NT3	Neurotrophin-3
ORA	Over-Representation Analysis
PFA	Paraformaldehyde
SPF	Specific Pathogen-Free
TFs	Transcription Factors
TMZ	Temozolomide
TGF-β	Transforming Growth Factor Beta
UMAP	Uniform Manifold Approximation and Projection
VPA	Valproic Acid
