# Supplementary material for: Small Molecule Cocktail DLC79 Suppresses Gliomagenesis by Activating Ascl1 and Remodeling Transcriptome
Source: Cells. 2026 Jan 22;15(2):211. doi: 10.3390/cells15020211 (PMC12839642; doi:10.3390/cells15020211)
Supplement: Supplementary file 1 [file cells-15-00211-s001.zip › cells-4061062-supplementary.pdf]

Supporting Information

Supporting Figures

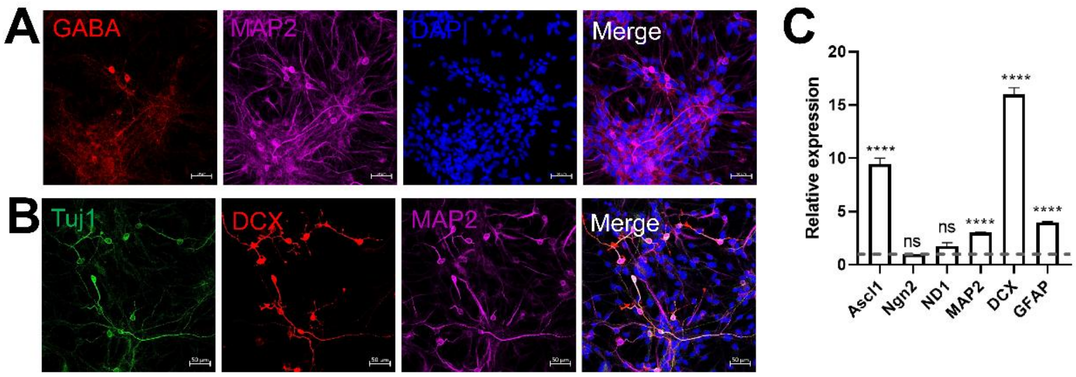

Figure S1. DLC79 Converted the Glioma Cells into Inhibitory Neuron Cells. (A) Immunofluorescence staining images of the neuronal subtype markers GABA and MAP2 in U251 cells induced by DLC79 for 24 days. (B) Immunofluorescence staining images of the neuronal markers Tuj1, MAP2, and DCX in U251 cells induced by DLC79 for 24 days. Scale bar 100 μm. (C) The gene expression in U251 cells induced by DLC79 for 40 days compared to the control group. The ns indicates no significant difference. Data are represented as mean ± SEM. ****$p < 0.0001$, Student's t-test, n=3.

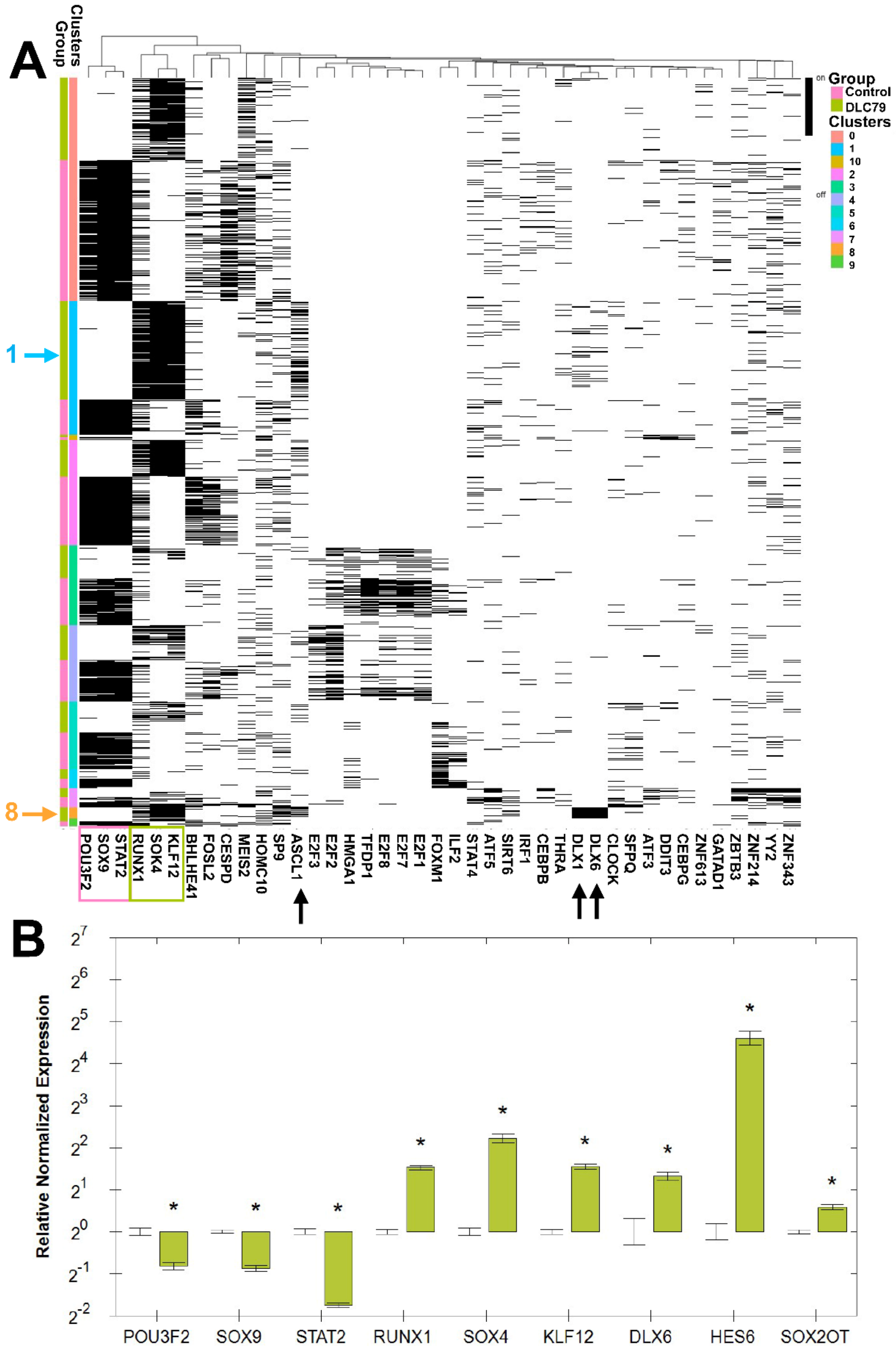

Figure S2. DLC79 Triggered the Activation of Neuronal Transcription Factors. (A) Single-cell regulatory network inference and clustering (SCENIC, version 1.1.2.1) results of the DLC79-treated group and the control group. Transcription factors highly expressed in the control group are indicated

within the red boxes, while those upregulated after DLC79 treatment are marked with green boxes. Black arrows highlight key neuronal transcription factors associated with GABAergic neuron differentiation. The arrows on the left indicate highly expressed transcription factors in cluster 1 and cluster 8, respectively. (B) Quantitative PCR results of the relevant transcription factors shown in (A), presented as fold changes in the DLC79-treated group relative to the control group after 4 days of treatment. * $p < 0.05$, Student's t-test, n=3.

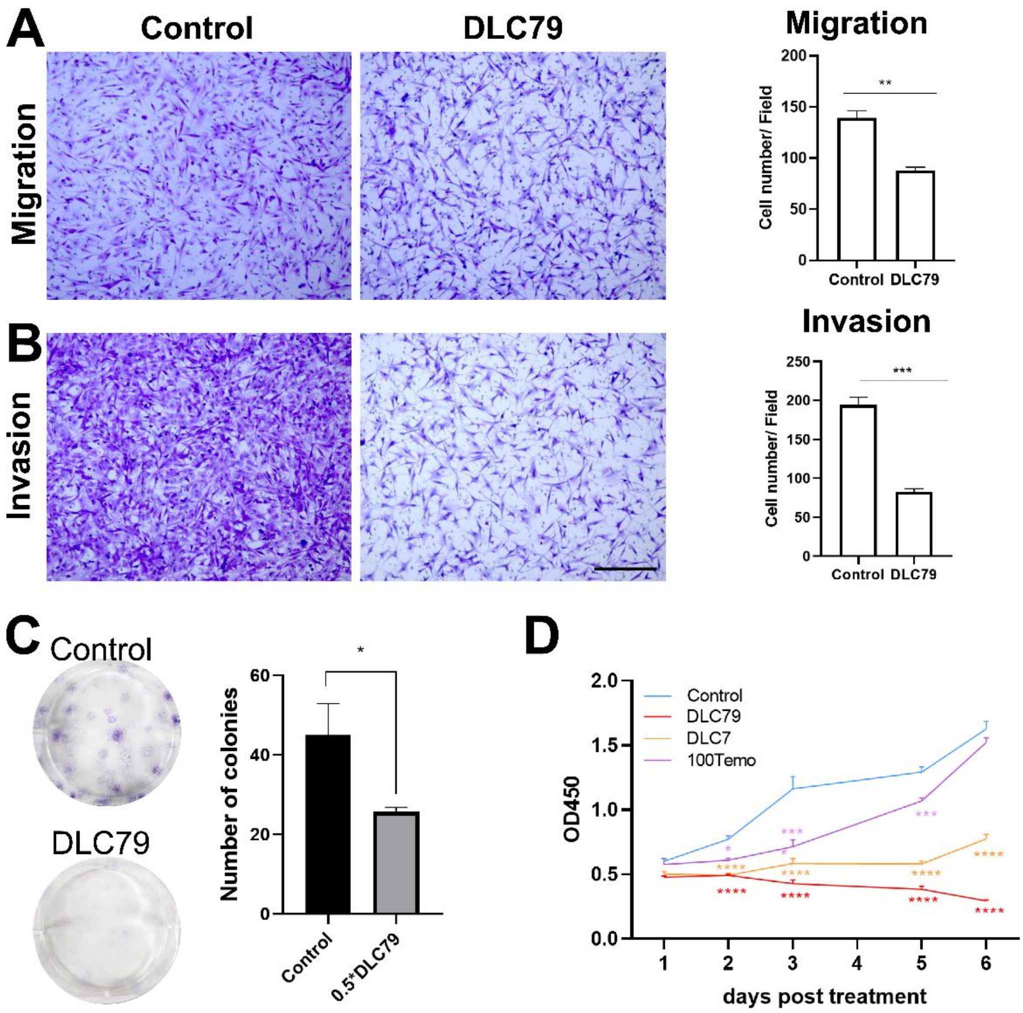

Figure S3. DLC79 reduces malignant features of U87-Luci cells in vitro. (A) Representative pictures and quantification data showing U87-Luci migration reduced after DLC79 treatment. Scale bar. ** $p < 0.01$. (B) Representative pictures and quantification data showing U87-Luci invasion reduced after DLC79 treatment. Scale bar. *** $p < 0.001$. (C) Colony formation assay and quantification data showing DLC79 treatment inhibits colony formation ability of U87-Luci followed by DLC7 and 100 μM TMZ as a control. One-way ANOVA with Tukey's multiple comparisons. Data

are represented as mean ± SEM, * $p < 0.05$. (D) Quantitative data of CCK-8 assay showing DLC79 treatment inhibits glioma cell proliferation with a lowest OD_{450} value followed by DLC7 and 100 μM TMZ as a control. Two-way ANOVA with Tukey's multiple comparisons. Data are represented as mean ± SEM, * $p < 0.01$, *** $p < 0.001$, **** $p < 0.0001$.

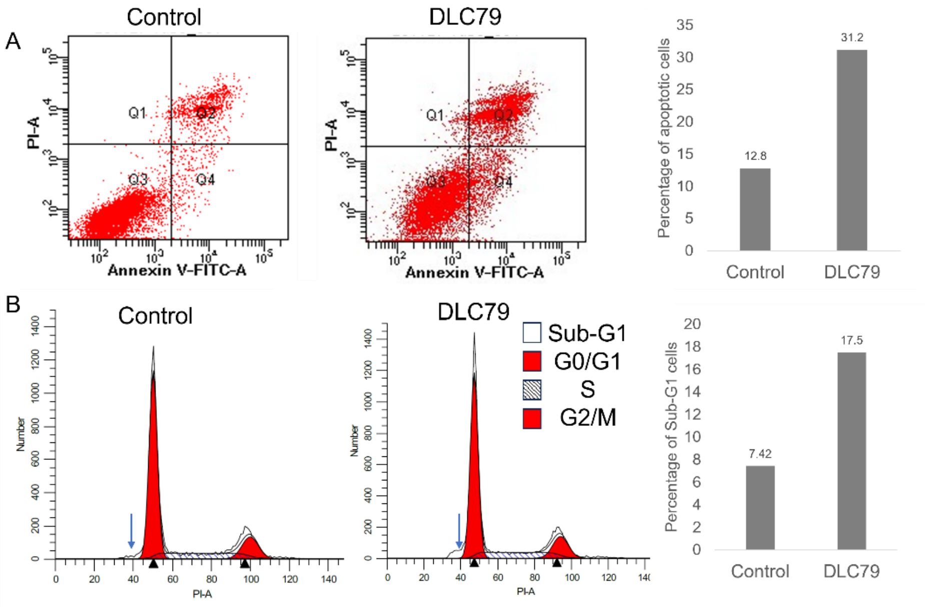

Figure S4: Changes in apoptosis and cell cycle on Day 2 of DLC79 treatment in U251 cells. (A) The Annexin V/PI staining to quantify apoptosis, results showing that DLC79 treatment significantly increased the early apoptosis rate to 31.2%, compared to 12.8% in the DMSO control group. (B) DLC79 treatment induced a marked increase in the Sub-G1 population in DLC79 17.5%, while 7.42% in control.

Table S1. The list of all the antibodies.

Antigen	Host species	Clonality	Manufacturers	Catalog number	Dilution
GFAP	Rat	Polyclonal	Invitrogen	13-0300	1:1000
DCX	Rabbit	Polyclonal	Abcam	Ab18723	1:2000
Tuj1	Mouse	Monoclonal	Sigma	T8660	1:1000
MAP2	Chicken	Polyclonal	Abcam	Ab5392	1:2000
GABA	Rabbit	Polyclonal	Sigma	A2052	1:1000
Mash1/Ascl1	Rabbit	Monoclonal	Abcam	ab211327	1:1000
Neurog2	Rabbit	Polyclonal	Invitrogen	PA5-78556	1:1000
NeuroD1	Rabbit	Monoclonal	Abcam	ab205300	1:1000
vGlut1	Rabbit	Polyclonal	SYSY	135302	1:1000
Ki67	Rat	Monoclonal	Invitrogen	14-5698-82	1:1000
SOX2	Rabbit	Polyclonal	Millipore	Ab5603	1:1000
SOX9	Rabbit	Polyclonal	Millipore	Ab5535	1:1000
S100B	Mouse	Monoclonal	Sigma	S2532	1:1000
NeuN	Rabbit	Monoclonal	Millipore	ABN78	1:1000
Alexa Fluor 488	Donkey anti mouse	Polyclonal	Invitrogen	A21202	1:1000
Alexa Fluor 488	Goat anti chicken	Polyclonal	Invitrogen	A11039	1:1000
Alexa Fluor 555	Donkey anti rabbit	Polyclonal	Invitrogen	A31572	1:1000
Alexa Fluor 546	Goat anti chicken	Polyclonal	Invitrogen	A11040	1:1000
Alexa Fluor 647	Donkey anti mouse	Polyclonal	Invitrogen	A31571	1:1000
Alexa Fluor 647	Goat anti rat	Polyclonal	Invitrogen	A21247	1:1000

Table S2. The targets and functions of the screened small molecules.

Small molecules	Targets and Functions	Ref
SB431542	A TGF-β receptor kinase inhibitor.	[24]
DAPT	A potent and orally active γ-secretase inhibitor. DAPT inhibits the activation of Notch signaling and induces cell differentiation.	[24]
CHIR99021 (Laduviglusib)	A potent, selective and orally active GSK-3α/β inhibitor. CHIR99021 is also a potent Wnt/β-catenin signaling pathway activator.	[24]
LDN193189	A potent selective BMP type I receptor (BMP I) inhibitor.	[24]
I-BET151	A BET bromodomain inhibitor which inhibits BRD4, BRD2, and BRD3.	[25-27]
I-BET762 (Molibresib)	I-BET762 (Molibresib) a BET bromodomain inhibitor	[29]
Isx9	A potent inducer of adult neural stem cell differentiation. ISX-9 activates Ca^{2+} influx through both voltage-gated Ca^{2+} channels and NMDA receptors and increases neuroD expression.	[28]
Dihydromyricetin	A potent inhibitor on dihydropyrimidinase. Dihydromyricetin can activate autophagy through inhibiting mTOR signaling.	[41]
SKL2001	An agonist of the Wnt/β-catenin pathway, with anti-cancer activity.	[42]
Repsox	A potent and selective transforming growth factor-beta receptor I /activin like kinase 5 (TGF-β-RI/ALK5) inhibitor.	[22]
Forskolin (Coleonol)	A potent adenylate cyclase activator. Forskolin is also an inducer of intracellular cAMP formation.	[43]
Purmorphamine	A smoothened/Smo receptor agonist.	[22]
SAG	A potent Smoothened (Smo) receptor agonist. SAG activates the Hedgehog signaling pathway and counteracts Cyclopamine inhibition of Smo.	[22]
Cyclopamine	A Hedgehog (Hh) pathway antagonist. Cyclopamine is also a selective Smo inhibitor.	[44]
Y27632	An orally active, ATP-competitive inhibitor of ROCK-I and ROCK-II.	[45]
Dorsomorphin (Compound C)	A selective and ATP-competitive AMPK inhibitor. Dorsomorphin selectively inhibits BMP type I receptors ALK2, ALK3, and ALK6.	[46]
Valproic acid (VPA)	An orally active HDAC inhibitor. Valproic acid inhibits HDAC1, and induces proteasomal degradation of HDAC2.	[22]
Metformin	Metformin regulates the expression of autophagy-related proteins by activating AMPK and inhibiting the mTOR signaling pathway, thereby inducing tumor cell autophagy.	[47]
Berberine (Natural	An alkaloid isolated from the Chinese herbal medicine Huanglian,	[48]

Yellow 18)	as an antibiotic. Berberine induces reactive oxygen species (ROS) generation and inhibits DNA topoisomerase. Berberine has antineoplastic properties.	
Temozolomide (NSC 362856)	An oral active DNA alkylating agent that crosses the blood-brain barrier. Temozolomide is also a proautophagic and proapoptotic agent. Temozolomide is effective against tumor cells that are characterized by low levels of O6-alkylguanine DNA alkyltransferase (OGAT) and a functional mismatch repair system.	[49]

Table S3. Top 3 highly expression gene along significantly changed clusters in single cell sequence

Clusters	Gene symbol	Description
Cluster 0	*AC021134.1*	long non-coding RNA
	NPY1R	Neuropeptide Y receptor Y1
	MEST	Mesoderm Specific Transcript
Cluster 2	*CCN1*	cellular communication network factor 1
	RUNX2	RUNX Family Transcription Factor 2
	FABP7	Fatty Acid Binding Protein 7
Cluster 3	*HIST2H2AC*	Histone H2A type 2-C
	HIST1H1C	Histone Cluster 1 H1 Family Member C
	HIST1H4C	Histone H4
Cluster 1	*RGS16*	Regulator of G Protein Signaling 16
	SCG2	Secretogranin II
	LSAMP	Limbic System Associated Membrane Protein
Cluster 8	*DCX*	Doublecortin
	DLX1	Distal-Less Homeobox 1
	DLX6-AS1	Distal-Less Homeobox 6 Antisense RNA 1
